# Effect of commercially purified deoxynivalenol and zearalenone mycotoxins on microbial diversity of pig cecum contents

**DOI:** 10.5713/ajas.20.0137

**Published:** 2020-05-12

**Authors:** Kondreddy Eswar Reddy, Minji Kim, Ki Hyun Kim, Sang Yun Ji, Youlchang Baek, Ju Lan Chun, Hyun Jung Jung, Changyong Choe, Hyun Jeong Lee, Minseok Kim, Sung Dae Lee

**Affiliations:** 1 Animal Nutrition and Physiology Team, National Institute of Animal Science, Rural Development Administration, Wanju 55365, Korea; 2 Swine Science Division, National Institute of Animal Science, Rural Development Administration, Cheonan 31000, Korea; 3 Division of Animal Disease and Health, National Institute of Animal Science, Rural Development Administration, Wanju 55365, Korea; 4 Dairy Science Division, National Institute of Animal Science, Rural Development Administration, Cheonan 31000, Korea; 5 Department of Animal Science, College of Agriculture and Life Science, Chonnam National University, Gwangju 61186, Korea

**Keywords:** Mycotoxins, Pig, Intestine, Deoxynivalenol, Zearalenone, Detoxification

## Abstract

**Objective:**

Deoxynivalenol (DON) and zearalenone (ZEN) are mycotoxins that frequently contaminate maize and grain cereals, imposing risks to the health of both humans and animals and leading to economic losses. The gut microbiome has been shown to help combat the effects of such toxins, with certain microorganisms reported to contribute significantly to the detoxification process.

**Methods:**

We examined the cecum contents of three different dietary groups of pigs (control, as well as diets contaminated with 8 mg DON/kg feed or 0.8 mg ZEN/kg feed). Bacterial 16S rRNA gene amplicons were acquired from the cecum contents and evaluated by next-generation sequencing.

**Results:**

A total of 2,539,288 sequences were generated with ~500 nucleotide read lengths. Firmicutes, Bacteroidetes, and Proteobacteria were the dominant phyla, occupying more than 96% of all three groups. *Lactobacillus*, *Bacteroides*, *Megasphaera*, and *Campylobacter* showed potential as biomarkers for each group. Particularly, *Lactobacillus* and *Bacteroides* were more abundant in the DON and ZEN groups than in the control. Additionally, 52,414 operational taxonomic units were detected in the three groups; those of *Bacteroides*, *Lactobacillus*, *Campylobacter*, and *Prevotella* were most dominant and significantly varied between groups. Hence, contamination of feed by DON and ZEN affected the cecum microbiota, while *Lactobacillus* and *Bacteroides* were highly abundant and positively influenced the host physiology.

**Conclusion:**

*Lactobacillus* and *Bacteroides* play key roles in the process of detoxification and improving the immune response. We, therefore, believe that these results may be useful for determining whether disturbances in the intestinal microflora, such as the toxic effects of DON and ZEN, can be treated by modulating the intestinal bacterial flora.

## INTRODUCTION

Deoxynivalenol (DON) and zearalenone (ZEN) are mycotoxins produced by *Fusarium* fungi, which frequently contaminate maize and grain cereals [1]. These mycotoxins have been widely investigated due to their universal distribution and capacity to cause pathological changes in humans and farm animals. Mycotoxicosis is the term used to refer to diseases caused by these toxins in humans and animals, often presenting in farm animals as reduced feed intake, poor feed conversion, feed refusal, reduced body weight gain, immune suppression, poor reproductive capacity, and long-term chronic effects, ultimately resulting in economic losses.

Numerous mechanisms have been suggested to explain the biological effects elicited by DON-contaminated feed. For instance, long-term exposure to DON may cause anorexia, reduced feed intake and weight gain, decreased nutritional efficiency, and immune modulation [2,3]. According to Pestka and Smolinski [4], DON inhibits protein biosynthesis and induces pro-inflammatory cytokine production. Among farm animals, pigs are the most sensitive to DON with chronic exposure, characterized as 1 to 2 mg DON/kg in feed, resulting in decreased appetite; whereas 3 mg DON/kg reduces body temperature and induces variations in the gastric wall of piglets. Further, according to Reddy et al [3], consumption of a diet containing 8 mg DON/kg for four weeks decreased body weight gain, reduced feed conversion rate, affected inflammatory cytokine production, reduced immunoglobulin (Ig)G, IgM, and serotonin values, and decreased total antioxidant contents in serum samples of pigs, as well as histopathological damage in kidney and liver samples compared to a standard dietary group. Furthermore, the usual growth rate of pigs has been reported to be reduced by approximately 7% for each mg of DON/kg increase in the diet, which may vary depending on numerous factors. Meanwhile, complete refusal of feed occurs at concentrations greater than 12 mg DON/kg. According to Young et al [5], high doses of DON (0.1 to 0.3 mg/kg body weight or 20 mg/kg of feed) in piglets led to vomiting. When pigs consume DON it becomes absorbed in the proximal aspect of the small intestine; and once in the colon de-epoxidation can occur, however, detoxification is not significantly induced [6].

The ZEN is a phytoestrogenic compound with estrogenic effects in farm animals [7]. The ZEN is associated with various dose-dependent mycotoxicoses in farm animals, particularly pigs. Pigs generally show clinical signs at low doses of ZEN (1.5 to 2 mg/kg in diet) including vaginal and vulvar swelling and thickness, increased uterus mass, testicular atrophy, and expansion of the mammary glands, as well as other reproductive effects, such as decreased fertility, increased number of resorptions, and reduced litter size [7]. Typically, hyperestrogenism and mortality are observed in pigs fed high doses of ZEN over an extended period of time [8], and infertility was observed at concentrations higher than 100 mg ZEN/kg [9]. A recent study [3] showed that in pigs fed a 0.8 mg ZEN/kg diet for four weeks, the serum IgG and IgM levels decreased; meanwhile total antioxidant levels decreased in the serum yet increased in urine. At the same ZEN dose, inflammatory cytokine and chemokine marker expression was reduced in the kidney and liver tissues, with microscopic lesions appearing in these tissues.

The gastrointestinal (GI) microbiota plays essential roles in animals, with a strong association reported between the host and its GI microbiota, particularly in immune response, energy uptake from food, activity of important metabolites, resistance to toxins, and metabolic products of fermentation. However, few studies have examined the GI microbiota in farm animals [10], particularly the effects of DON and ZEN mycotoxins on the pig intestinal microbiota communities. Different species colonize various areas of the GI tract to different degrees. Pigs, specifically, are highly sensitive to dietary mycotoxins, which target the mucus and microbiota [11]. For instance, *Fusarium* toxins can cause gut tissue damage, shorten the height of villi, and cause dysbiosis of gut microbiota [12]. We previously showed that *Fusarium* mycotoxins primarily influence the composition of pig gut microbiota, ultimately causing activation of intestinal inflammation [10].

Among the various gut microbiota, bacteria play a major role in the GI tract. Next-generation DNA sequencing (NGS) approaches are effective for studying the composition of the host microbiota, with the high read abundance of amplified sequences providing insight into microbial diversity. Advanced molecular biology techniques can be useful in examining complex microbiological communities, such as those in the gut that cannot be cultured outside of the host. Moreover, these approaches have revealed variations in the gut bacterial community between functional sites, individuals, as well as healthy and diseased conditions [13]. Importantly, studies of the gut microbiota have also demonstrated its critical role in maintaining animal and human health.

The direct effects exerted by consumption of highly concentrated and commercially-purified DON and ZEN on the composition of the cecum microbiota have not been reported in pigs. Therefore, this study was performed to determine the effect of commercially-purified *Fusarium* mycotoxins, DON and ZEN, on individual pigs after four weeks of feeding, as well as to compare mycotoxin-treated pig groups to gain insight into the quantitative and qualitative composition of the pig cecum microbiota. These results may be useful for determining whether disturbances in the intestinal microflora, such as the toxic effects of DON and ZEN, can be ameliorated via modulating the intestinal bacterial flora.

## MATERIALS AND METHODS

### Ethics statement

The protocols for the animal experimental procedures were reviewed and accepted by the Institutional Animal Care and Use Committee of the National Institute of Animal Science, South Korea (No. 2015–147).

### Experimental design and animal exposure to DON and ZEN

This study was conducted using 14 male castrated 8-week-old piglets (Landrace×Yorkshire = Large White Landrace; ~19 kg), obtained from a commercial pig farm. Each pig was housed in an individual pen (2.1×1.4 m) at 25°C±1°C. The pigs were allowed to adjust to their new housing conditions for 1 week, and then assigned to three dietary groups, control (n = 4), DON (n = 5), and ZEN (n = 5) groups, with approximately equal body weights. The control group was fed a standard diet to meet the nutritional requirements for piglets (Table 1) [14], whereas the two treatment groups were fed a standard diet plus DON (8 mg/kg) or ZEN (0.8 mg/kg) for 4 weeks. The commercial DON and ZEN powders were added as purified toxins (Biomin Pte. Ltd., Singapore) and properly mixed into the diet at 8 and 0.8 mg/kg, respectively. The control feeds, DON-and ZEN-purified mycotoxins contaminated feeds, and water were provided *ad libitum* throughout the 4-week experimental period.

### Mycotoxins analysis from the feed

The DON and ZEN in the supplemented corn feeds were quantitatively evaluated by ultra-performance liquid chromatography (UPLC). Briefly, a homogenized DON- or ZEN-mixed grain sample (1 g) was extracted with 20 mL of distilled water and shaken for 30 min (DON samples) or with 0.5 g of NaCl and 20 mL acetonitrile and shaken for 1 h (ZEN sample). After filtering the extract through Whatman paper (No. 1), 5 mL of the DON filtrate sample was diluted in 20 mL of phosphate-buffered saline, and 5 mL of the ZEN filtrate sample was diluted in 20 mL of 1% Tween 20 solution. The extracted DON and ZEN samples were loaded separately onto immunoaffinity chromatography columns. UPLC methods and mass spectrometry were performed as described previously by Reddy et al [15]. The limit of detection (LOD) was 5 μg/kg for both DON and ZEN, and the limit of quantification was 10 μg/kg for DON and 16.7 μg/kg for ZEN. Analysis of the standard diet as well as the DON and ZEN-contaminated feed was repeated three times. We found that the total amount of DON and ZEN in the mixed corn feed was 7.38 mg/kg and 0.67 mg/kg feed, respectively, which are similar to that of the original concentrations of mixed corn feed. Alternatively, the control diet was free of mycotoxins or it may have simply contained levels below the LOD.

### Sampling and processing

A total of 14 castrated male piglets were used to study the cecum microbiota composition in the standard diet and DON and ZEN mycotoxin-contaminated feed groups. After 4 weeks, standard diet pigs and pigs fed a diet containing DON and ZEN were sacrificed by anesthetic pentobarbital overdose. Following complete cardiac arrest, the cecum contents of all pigs were aseptically collected directly into sample containers, quickly frozen in liquid nitrogen, and stored at −80°C until analysis.

### DNA extraction and 16S rRNA gene sequencing

DNA was extracted from the pig cecum contents of each group by the repeated bead beating plus column method [16], and stored at −20°C. From each DNA sample, 14 amplicon libraries were produced using the 341F (5′-CCTACGGGNGGCWG CAG-3′) and 805R (5′-GACTACHVGGGTATCTAATCC -3′) primers, which typically produce approximately 450-base pair products. The resulting 14 amplicon libraries were constructed using a paired-end protocol with the MiSeq platform (Illumina, San Diego, CA, USA) at the Macrogen sequencing facility (Macrogen, Inc., Seoul, Korea). Paired reads were collected using the FLASH program [17]. The assembled sequences were demultiplexed using the default parameters and quality filtered using a Q20 minimum value with the QIIME software package 1.9.1 [18]. According to DeSantis et al [19], the taxa were determined using the Greengenes reference database, while operational taxonomic units (OTUs) were determined at 97% sequence similarity using the uclust program [20]. After 100,000 sequences were subsampled from each cecum sample to normalize the number of OTUs, alpha diversity indices, including the number of OTUs, Chao1, PD_whole_tree distance, and Shannon diversity index were determined.

### Statistical analysis

Taxa (or OTUs) accounting for an average of ≤0.2% of the total sequences were considered as major taxa (or OTUs) and statistically analyzed. The taxonomic composition of each taxon or OTU in the total sequences was compared among the control, DON, and ZEN dietary groups by analysis of variance, followed by Duncan’s test using XLSTAT statistical software version 18.07 (Addinsoft, New York, NY, USA). A significant difference was considered at p<0.05.

## RESULTS

### Sequencing and bacterial abundance

Quality control of all cleaned 16S rRNA sequencing (*rrs*) reads among the pig cecum contents in the control, DON, and ZEN dietary treatment groups resulted in identification of 2,539,288 sequences with ~500-nucleotide read lengths. From these group sequences, 714,639 represented control dietary group cecum contents, whereas 890,094 and 934,555 represented DON and ZEN dietary treated cecum contents, respectively. The numbers of *rrs* sequences from individual control dietary cecum samples were 171,037 to 187,601; those from the DON-dietary treated cecum samples were 155,260 to 189,289, and those from ZEN-dietary treated samples were 146,729 to 212,732.

**Figure 1:**
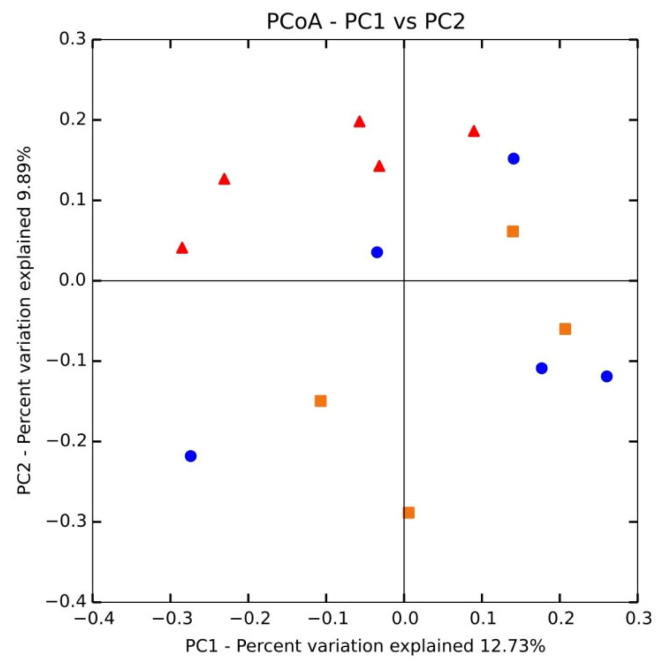
Principal coordinates analysis (PCoA) of cecum microbial communities after different dietary treatments. PCoA based on the weighted UniFrac distance of 16s rRNA samples of cecum bacteria in the control (orange dots), DON (red dots), and ZEN (blue dots) dietary treatment groups. DON, deoxynivalenol; ZEN, zearalenone.

We used principal coordinate analysis (PCoA; Figure 1) with weighted uniFrac distances to compare the sample structures. Most DON samples showed an anterior distribution and maintained a certain distance from the posterior control and ZEN dietary samples. Two ZEN-treated samples were nearer to DON and clearly separated from the control group. These results suggest intra-group variation.

### Bacterial taxonomic composition

**Figure 2:**
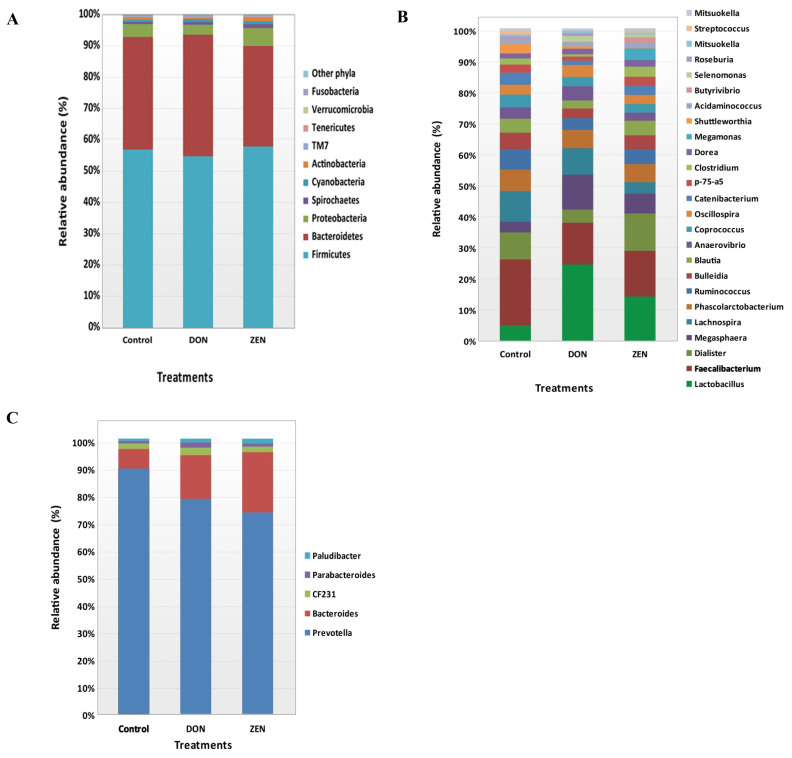
Relative abundances of microbial taxa in the cecum at the phylum and genus levels in the control, deoxynivalenol (DON), and zearalenone (ZEN) dietary treatment groups. (A) Relative abundances of taxa within all phyla. (B) Abundance of Firmicutes. (C) Abundance of Bacteroidetes.

All *rrs* sequences from the cecum contents of the control, DON, and ZEN dietary groups differed at the phylum, family, and genus levels (Figure 2A, 2C, Supplementary Table S1). The microbial data for all three dietary groups were examined to determine the mean relative abundance (taxon reads/total reads in a sample). The phyla Firmicutes and Bacteroidetes were the most abundant in the three groups, accounting for 56.8% and 35.6% of all *rrs* sequences, respectively (Figure 2A). The third richest phylum was Proteobacteria, accounting for 4.3% of the total sequences in the three groups. The remaining identified phyla showed <1.0% abundance of all *rrs* sequences and included Spirochetes (0.9%) Cyanobacteria (0.9%), Actinobacteria (0.8%), TM7 (0.4%), Tenericutes (0.3%), Verrucomicrobia (0.2%), and Fusobacteria (0.1%) (Supplementary Table S1). Significant differences were found only in the phylum Verrucomicrobia among the dietary treatment groups.

As shown in Table 2, at the family level, Prevotellaceae was highly abundant, accounting for 21.4% of the collective data; the remaining most abundant families were Ruminococcaceae (14.7%), Lachnospiraceae (13.6%), Veillonellaceae (11.1%), Bacteroidaceae (4.1%), Lactobacillaceae (4.0%), Erysipelotrichaceae (3.8%), and Clostridiaceae (2.0%). The other 21 families detected represented <2.0% of all dietary group sequences (Table 2). Among the families, Bacteroidaceae (4.1%), Lactobacillaceae (4.0%), Clostridiaceae (2.0%), Campylobacteraceae (1.4%), Aeromonadaceae (0.5%), Chlamydomonadaceae (0.4%), Pasteurellaceae (0.3%), Turicibacteraceae (0.2%), Rhodocyclaceae (0.2%), Verrucomicrobiaceae (0.1%), Christensenellaceae (0.1%), and Flavobacteriaceae (0.1%) showed significant differences (p<0.05) among the dietary treatment groups. Most families with significant differences were more abundant in the DON and ZEN treatment groups than in the control. Particularly, Lactobacillaceae showed a high abundance in the DON dietary group (6.5%) and ZEN dietary group (3.8%) compared to the control group (1.1%). Bacteroidaceae also showed a high abundance in both the DON and ZEN dietary groups (4.6% and 5.3%, respectively) compared to the control (2.0%). However, Clostridiaceae showed high abundance in the control (3.0%) and ZEN (2.7%) dietary groups compared to in the DON group (0.4%). Similarly, Campylobacteraceae was abundant in the control (1.5%) and ZEN (2.8%) groups, whereas this family was not detected in the DON dietary group. The families Clostridiaceae, Pasteurellaceae, and Turicibacteraceae families showed high abundance in the ZEN group and very low abundance in the DON group compared to in the control group.

**Figure 3:**
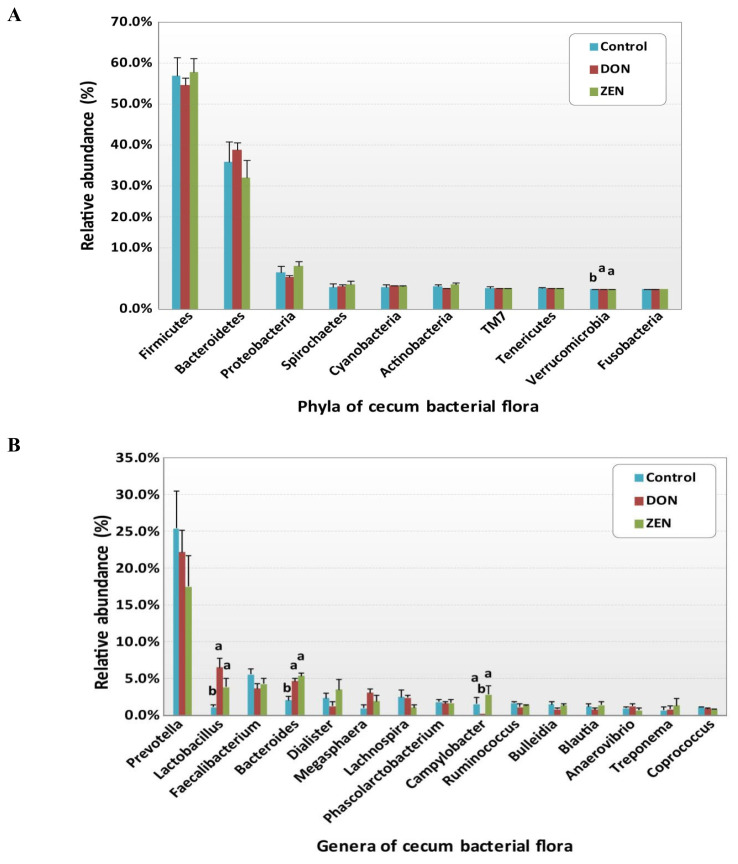
Relative abundances of the cecum microbiota among the control and deoxynivalenol (DON) and zearalenone (ZEN) mycotoxin groups. (A) Variations in the relative abundance of the cecum microbiota at the phylum level. (B) Differences in the relative abundance of the top 15 cecum bacteria at the genus level. Different letters indicate significant differences (p<0.05).

Variations in the abundance of the ten phyla between the dietary treatments are shown in Figure 3A, and the top 15 genera with the highest richness variation between the control, DON, and ZEN dietary treatment groups are shown in Figure 3B. *Prevotella* was highly abundant at the genus level in the three dietary groups accounting for 21.4% of the 2,539,288 *rrs* sequences. Other genera, represented by at least 0.5% of the total three dietary group sequences, were *Faecalibacterium* (4.4%) *Bacteroides* (4.1%), *Lactobacillus* (4.0%), *Dialister* (2.3%), *Megasphaera* (2.0%) *Lachnospira* (1.9%) *Phascolarctobacterium* (1.7%), *Campylobacter* (1.4%), *Ruminococcus* (1.3%), *Bulleidia* (1.2%), *Blautia* (1.1%), *Anaerovibrio* (0.9%), *Coprococcus* (0.9%), *Oscillospira* (0.9%), *Treponema* (0.9%), *Catenibacterium* (0.7%), *p-75-a5* (0.6%), CF231 (0.6%), and *Clostridium* (0.5%). Differences in the abundance of phyla among the dietary groups were observed; however, few of these differences were significant between the control and treatment groups (Supplementary Table S1).

The phylum Firmicutes was most abundant and collectively occupied 56.8% of phyla in the three dietary groups, with values of 56.7%, 54.07%, and 57.8% in the control, DON, and ZEN groups, respectively (Supplementary Table S1). Twenty-six genera within Firmicutes were detected, which comprised at least 0.2% of all sequences in each dietary group; mainly, *Faecalibacterium* (4.4%) and *Lactobacillus* (4.0%) showed high abundance compared to the other genera. *Faecalibacterium* was detected at 5.5%, 3.6%, and 4.2% in the control, DON, and ZEN dietary groups, respectively, with no significant difference between groups. The collective data of *Lactobacillus* abundance was 4.0%, with significant differences (p = 0.005) observed between the dietary treatments. Interestingly, *Lactobacillus* levels were nearly six-fold higher in the DON (6.5%) group, and more than three-fold higher in the ZEN (3.8%) group compared to in the control (1.1%). *Megasphaera* also showed significantly high abundance (p = 0.05) and comprised 2.0% of all sequences, with higher abundance in the DON (3.0%) and ZEN (1.8%) treatment groups than in the control (0.9%). *Peptococcus* and *Pelosinus* also showed significant differences (p<0.05) among the dietary treatments. *Peptococcus* showed 0.2% abundance in the control and 0.1% in ZEN, with no abundance in DON (0.0%); whereas *Pelosinus* showed 0.2% abundance in the DON group, 0.1% in the ZEN group, and 0.0% in the control group. The major Firmicutes species identified were *Faecalibacterium prausnitzii* (4.4%), *Ruminococcus bromii* (0.2%), and *Selenomonas ruminantium* (0.2%), which did not differ (p>0.05) in abundance between the three dietary groups.

The phylum Bacteroidetes was second most abundant and collectively occupied 35.6% of the total sequences. Bacteroidetes showed 35.9%, 38.8%, and 32.0% in the control, DON and ZEN dietary treatments, respectively. In Bacteroidetes, the most abundant genera were *Prevotella*, *Bacteroides*, *CF231*, *Parabacteroides*, and *Paludibacter*. *Prevotella* showed high abundance with 25.4% and 22.2% in the control and DON groups, with a low abundance of 17.5% in the ZEN group (p>0.05). *Bacteroides* showed high abundance (4.1%) and significant differences (p = 0.005) among the dietary groups, at 2.0% in the control group; the DON (4.6%) and ZEN (5.3%) dietary groups showed more than two-fold higher levels compared to the control group. *Paludibacter* also showed significant differences between dietary groups, with both the DON and ZEN dietary groups exhibiting two-fold higher abundance (0.4%) compared to the control group (0.2%). The major Bacteroidetes species identified were *Prevotella copri* (10.7%) and *Prevotella stercorea* (1.5%), however, their abundance did not differ (p>0.05) among the three dietary groups.

Proteobacteria was the third most prevalent phylum (4.3%). Within this phylum, we found five genera: *Campylobacter*, *Succinivibrio*, *Actinobacillus*, *Desulfovibrio*, and *Dechloromonas*, of which *Campylobacter* showed significantly high abundance in the ZEN (2.8%) and control groups (1.5%), with no abundance in the DON group (0.0%). *Actinobacillus* and *Dechloromonas* also showed significant differences among the dietary groups as did *Turicibacter*, in the phylum Actinobacteria, and *Akkermansia*, in the phylum Verrucomicrobia.

### Analysis of operational taxonomic unit diversity

A total of 52,415 OTUs were found among the three dietary treatment group samples at a 0.03 dissimilarity cutoff level. All sequence reads were normalized and examined using the Shannon diversity index, Chao1 richness estimator, and Simpson index. The normalized samples did not vary in richness (p>0.05) for the numbers of OTUs among the three dietary treatment groups, however, significant differences were observed (p = 0.05) between the double sequence reads (Table 3). Seventeen of the 52,415 OTUs were significantly (p<0.05) represented by ≥0.2% of all sequences in at least one dietary treatment group, whereas seven of the 17 were classified into known genera, however, were not classified to any known species. The remaining ten OTUs could not be classified into known genera (Table 4). The major OTUs accounting ≥0.2% of all sequences were classified to species *Prevotella copri*, *Prevotella copri*, *Prevotella stercorea*, *Faecalibacterium prausnitzii*, or *Ruminococcus bromii*, however, their prevalence did not differ (p<0.05) among the three dietary treatment groups.

Among the 17 OTUs, *Bacteroides* (denovo163744) was the most dominant genera in the collective data, showing the highest prevalence in the DON and ZEN dietary groups and lowest in the control group. Similarly, another OTU also belonging to *Bacteroides* (denovo64865) was abundant in the DON and ZEN dietary groups, with low abundance in the control. *Lactobacillus* was the second most abundant OTU (denovo41137) and was much higher in the DON group, and moderately higher in the ZEN, compared to the control dietary group (Table 4). The next most abundant OTU was that representing the genus *Campylobacter* (denovo8890), which showed the greatest richness in the ZEN and control and lowest in the DON dietary groups. One OTU, classified as *Prevotella* (denovo47686), showed approximately five-fold higher abundance in ZEN and two-fold higher in DON compared to the control dietary group. The genus *Paludibacter* OTU (denovo77418) was also abundant in the DON and ZEN dietary groups compared to in the control group. The abundance of two unclassified Clostridiaceae OTU families (denovo3971 and denovo47258), and OTU Bulleidia genera (denovo116445) were highly prevalent in the control and ZEN dietary groups and lowest in the DON dietary treatment group. Three unclassified OTU families of Lachnospiraceae (denovo129399), Chlamydomonadaceae (denovo6037), and Aeromonadaceae (denovo48653) were much more abundant in both the DON and ZEN treatment groups compared to the control. Two other unclassified Lachnospiraceae OTU families (denovo24662 and denovo92328) showed significantly higher levels in the DON dietary treatment than in the ZEN and control groups. The abundance of unclassified OTUs Clostridiales (denovo14126), Erysipelotrichacea (denovo147371), and Ruminococcaceae (denovo153523), was much lower in the DON and ZEN treatment groups compared to the control.

## DISCUSSION

We assessed the effect of diets contaminated with DON and ZEN on the cecum microflora of pigs and evaluated which bacterial strains may reduce the toxicological effects of DON and ZEN. The GI microbiota in pigs has not been completely defined as this dynamic community contains many hundreds of species, comprised primarily of anaerobic bacteria [21]. The DON and ZEN mycotoxins have been shown to negatively affect GI microbiota, affected the GI species composition and bacterial numbers in pigs to varying degrees depending on the mycotoxin concentrations in diet, treatment period, animal age, location of toxins in the GI, and nutritional dietary factors. Although previous studies have examined the effects of feed naturally-contaminated with mycotoxins on the gut microbiota of pigs [22,23], here we sought to analyze the cecum contents of pigs fed commercially-purified DON and ZEN mycotoxins to compare microbial taxonomic abundances in the control, DON, and ZEN dietary treatment groups.

Among the phyla detected, Firmicutes (56.8%) and Bacteroidetes (35.6%) were highly abundant (Figure 3A), occupying more than 92% of the cecum contents from the DON, ZEN, and control dietary groups, however, their abundances showed no significant differences between dietary groups (Figure 2A, Supplementary Table S1). Similarly, in our previous study, the colons of DON and ZEN dietary-treated pigs also showed greater than 90% Firmicutes and Bacteroidetes (p>0.05) [10]. Typically, the compositions of the intestinal microbiota in the cecum and colon are similar among pigs [24]. However, Li et al [23] found that naturally DON-contaminated wheat fed to pigs caused differences in the abundance of Firmicutes and Bacteroidetes in the cecum, colon, and ileum. Further, Isaacson and Kim [24], evaluated naturally weaned pigs and found that Firmicutes and Bacteroidetes accounted for more than 90% of the bacteria detected in the cecum contents. Meanwhile, in another study, Firmicutes and Bacteroidetes comprised more than 90% of bacteria in pigs fed a fuminosin-contaminated diet (12 mg/kg feed) [25]. In the current study, no significant differences were noted at the phylum level in the toxic dietary treatment groups. However, if the dietary treatment period were to be extended, the microbiota may be altered in the toxin treatment groups. Family-level cecum bacterial abundances are shown in Table 2, and significant families in these genera were identified.

Among the cecum contents, specific genera were identified as potentially significant biomarkers for differentiating between the control and the DON and ZEN mycotoxin dietary treatment groups. Specifically, *Lactobacillus*, *Bacteroides*, and *Megasphaera* were significantly more abundant in the cecum microbiota of the DON and ZEN dietary groups compared to the control group (Figure 3b). *Lactobacillus* accounted for a predominant genus in DON and was moderately abundant in ZEN, meanwhile its levels were very low in the control group (Figure 2A). Similar results were observed in our previous study regarding the pattern of *Lactobacillus* abundance in the colons of pigs [10], with differences observed between the cecum and colon contents in the DON, ZEN, and control dietary treatments. It has been suggested that a myriad of factors contribute to the microbial shifts occurring between the cecum and colon, including stress resulting from mycotoxin-contaminated feed, chemical composition of the diet, as well as various physiological factors [26]. Recently, the abundance of *Lactobacillus* was found to be increased by 13% in the cecum of pigs fed DON- contaminated wheat in combination with *Clostridium* sp. WJ06 [23]. In another study, 15.8% of *Lactobacillus* sequences were recovered from the pig intestinal samples, indicating their important roles in the gut on host physiology [27]. Similarly, through 16S rRNA analysis, Niu et al [28] demonstrated that *Lactobacillus* is one of the most prevalent genera in pig intestinal samples, irrespective of age. Furthermore, NGS technology revealed *Lactobacillus* as a key member of the fecal microbiota in all growth stages of pigs.

*Lactobacillus* species are considered to be probiotics that play a key role in various physiological functions of their hosts, including microbial interference, antimicrobial properties, supplementary influences on nutrition, antitumor effects, decreasing cholesterol in the host serum, and immunomodulatory influences [29]. Particularly, in pigs, a positive effect was observed on *Lactobacillus* abundance following feed limitation. According to Yang et al [30], *Lactobacillus* can support the development of an optimized microbiome by enhancing the richness and number of lactobacilli and other native probiotic bacteria. The primary indigenous probiotic bacteria can promote growth and immunity of piglets through positive cascade signal transduction pathways. The piglet body provides a tolerant habitat and nutrients for bacterial colonization and growth, in return, probiotics generally generate prebiotics such as short-chain fatty acids and bacteriocins that can improve growth, and decrease the risk of enteric diseases caused by pathogens or toxins, while also enhancing the host feed utilization capacity. Furthermore, Walter [31] indicated that autochthonous *Lactobacillus* are often used as probiotics due to their natural capacity to survive harsh physiological conditions, including the acidic stomach, pancreatic enzymes, and bile salts during their passage through the GI tract [32,33]. These *Lactobacillus* probiotic bacteria were also selected for their capacity to adhere to mucus and epithelial cells, as these characteristics are required for their effective colonization of the gut’s mucosal and epithelial layer and to increase their competitiveness against pathogens [34]. This competitive profile is likely conferred by autochthonous *Lactobacillus*, which is used as a biomarker of health in the pig gut microflora.

According to many studies, the large quantity of lactic acid bacteria (LAB) in the digestive tract is associated with the age of pigs and feeding of probiotics. LAB play a crucial role in the host, generating substances such as acetic, butyric, and propionic acid, as well as other similar short-chain fatty acids, B vitamins, and amino acids, including bacteriocins ad antimicrobial metabolites. Colonization of the pig digestive tract by LAB is prevented by various pathogens stimulating the immune system of the host [35]. Moreover, LAB can bind *Fusarium* mycotoxins in their environment [36]. According to Franco et al [37], the capacity of viable and heat-inactivated *Lactobacillus* cells was reduced by more than 60% in the presence of 1.5 μg/mL DON in liquid media. However, according to Yang et al [38], *Lactobacillus* plays a key role in removing ZEN *in vitro*; meanwhile El-Nezami et al [39] reported the binding affinity of ZEN, and its derivative α-ZEN, with two food-grade strains of *Lactobacillus*. Still further, although the mechanisms of aflatoxin binding by specific *Lactobacillus* are unclear, cell wall peptidoglycans and polysaccharides have been suggested as the two most significant elements responsible for binding by *Lactobacillus* and the absorption of mutagens or carcinogens in the intestine [40]. In this study, we predicted that *Lactobacillus* concentrations were higher in DON and ZEN dietary treatment groups and that LAB may contribute significantly to the detoxification of DON and ZEN in the pig intestine. However, the intestinal mucus and its resident microbiota are important targets of dietary mycotoxins, particularly DON [11]. Consequently the GI mucus can reduce the ability of probiotics, such as *Lactobacillus*, to bind mycotoxins, thereby interfering with their adsorption of dietary mycotoxin. Similarly, in the current study, regular administration of the probiotic *Lactobacillus* during the dietary treatment period may have reduced the effect of mucus. The number of bacterial colony-forming units may have reduced the effects of mucus on the adsorption of DON and ZEN dietary mycotoxins by the *Lactobacillus* cell wall.

In the current study, *Bacteroides* accounted for another predominate genus, which was more abundant in the DON and ZEN dietary toxin groups than in the control. *Bacteroides* spp. are considered as a source of novel useful bacteria for treating gut immune dysfunctions, colitis, and metabolic disorders, as well as for cancer prevention [41]. *Bacteroides* spp. are dominant and play a key role in pig intestine, making them a major group in pig feces. Similar to our results, Saint-Cyr et al [42] demonstrated that feeding with DON at 100 mg/kg body weight for 4 weeks by oral gavage increased *Bacteroides* levels in rat intestines. Other studies in pigs supported the use of prebiotics for selecting *Bacteroides* spp. This capacity to influence the microbiota also involves inclusion of a specific level of fermentable fiber in the pig diet, which stimulates colonic fermentation, as well as the presence of exogenous enzymes, which create oligosaccharides with prebiotic effects from non-starch polysaccharides [43]. Moreover, *Bacteroides* spp. also help to protect against gut colonization by various potential pathogens. Based on these results, Bacteroides may play a key role in eradicating DON and ZEN from the pig gut. In this study, *Megasphaera* was approximately three-fold more abundant in the DON group, and two-fold higher in the ZEN group, compared to the control. Conversely in our previous study we reported that *Megasphaera* was more abundant in the ZEN dietary group, with no significant differences observed between the ZEN and control groups [10]. *Megasphaera* spp. are abundant and important in the human gut microbiota, and their capacity to produce important metabolites indicates beneficial health effects on the host. *Megasphaera elsdenii* was reported to comprise approximately 0.12% to 5.9% of bacteria in pig feces [44], where it uses both L- and _D_-lactate and increases short-chain fatty acids, which are crucial for pig colonocyte development and proliferation, as well as small intestine growth [45]. We, therefore, hypothesized that *Megasphaera* abundance increases in the intestines of pigs in response to the lactate produced by abundant *Lactobacillus*, and may positively influence intestinal disorders or immune responses following DON and ZEN dietary treatment of pigs. However, few studies have examined the effects of dietary mycotoxins on these bacteria in animals.

Other bacterial genera, including *Campylobacter*, *Paludibacter*, *Turibacter*, *Peptococcus*, *Pelosinus*, *Actinobacillus*, *Dechloromonas*, and *Akkermansia*, also showed significant differences between the dietary treatment groups. *Campylobacter* is the most common cause of GI infection in pigs, characterized by inflammation and diarrhea involving cramps, fever, and pain [46]. According to Burrough et al [47], *Paludibacter* is abundant in the pig intestine and proficiently ferments complex polysaccharides produced by the pig. We found that DON and ZEN influence the composition and fermentation products of the pig intestinal microbiota, thus affecting the health and performance of the pig. However, further studies are needed to determine the functions of these genera.

In the current study, 17 OTUs were identified that differed significantly in abundance between the control, DON, and ZEN dietary treatment groups. Similarly, our previous study of pig colon contents also showed varying OTUs abundances between these groups [10]. However, in the current study, most OTUs in the cecum differed from those in the colon. Specifically, *Bacteroides*, *Lactobacillus*, and *Campylobacter* OTUs were more abundant in the cecum than in the colon content; we also identified three unclassified Lachnospiraceae, and two unclassified Clostridiaceae family OTUs which differed in abundance between the DON and ZEN dietary groups. According to Przybylska-Gornowicz et al [12], the response of the intestinal immune system is unambiguous in the cecum; however, variable and sometimes difficult to interpret results were obtained in the ascending colon and descending colon. Unclassified Lachnospiraceae showed higher abundance in both DON and ZEN dietary treatment groups. Moreover, similar to our results, Gratz et al [48] found that the abundance of Lachnospiraceae was higher in the cecum contents of DON dietary pigs; no previous studies evaluated ZEN dietary pig treatment groups. Lachnospiraceae also showed higher abundance in fecal samples from pigs fed fumonisin in the diet [25]. Lachnospiraceae bacteria may also play a key role in healthy pigs and serve to improve the pig immune system. Compared to the control, unclassified Clostridiaceae was less abundant in DON, however, did not exhibit differences in abundance with the ZEN dietary group. Compared to that reported previously for the colon contents, here we demonstrate that DON OTU abundance was decreased in the cecum, while ZEN did not differ from the control group. According to Piotrowska et al [22], *Clostridium* abundance is reduced in the colons of both the DON and ZEN dietary groups in gilts, suggesting that the abundance of Clostridiaceae bacteria was inhibited by the highly toxic DON, with no significant effects imposed by ZEN, which has lower toxicity. *Prevotella* OTU was also significantly decreased in the DON group, without significant differences observed in the ZEN group compared to the control. Conversely, in our previous study we reported significantly higher OTU abundances in both the DON and ZEN dietary groups [15], with a higher *Prevotella* abundance in pigs fed fumonisin (12 mg/kg feed). The *Prevotella*-driven enterotype appears to be important in subjects who consumed high levels of carbohydrates and fiber. In this study, due to the high concentrations of DON and ZED, the daily feed intake was reduced in the DON group compared to in the ZEN and control groups, which subsequently reduced the abundance of *Prevotella* in the DON due to the reduced consumption of carbohydrates.

Since mycotoxins can alter the microbial composition balance of the pig intestine, a complete understanding of the relationship between pigs and their gut microbiota will facilitate the development of new dietary treatments that can increase pig growth, protect piglets from pathogenic bacteria, and enhance host feed utilization. In the current study, specific bacteria genera were highly abundant in both the DON and ZEN dietary treatments compared to in the control; in this case, this microbiota may have positively impacted the host physiology. Other bacteria in the DON and ZEN dietary treatments, particularly in the DON group, showed a lower abundance compared to the control; in these cases, DON and ZEN may have induced lesions in the cecum by disturbing the integrity of the pig intestinal barrier and reducing the abundance of specific microbiota. These results show that the composition and structure of the pig cecum greatly differs compared to that determined in our previous study of microflora in the pig colon. Similarly, a previous study also strongly demonstrated differences between cecum and colon microbial data in pigs [23]. DON and ZEN may alter susceptibility to infectious diseases in humans and animals by affecting gut health as well as the innate and adaptive immune systems. However, the mechanisms by which mycotoxins affect the intestinal microbiota composition remain unclear.

In conclusion, the results of this study indicate that the GI bacterial flora in pigs became disrupted following consumption of feed contaminated with commercial DON and ZEN for four weeks. The genera *Lactobacillus* (particularly in DON) and *Bacteroides* dominated the bacterial flora in both the DON and ZEN dietary treatments. In addition, OTUs assigned to unclassified Lachnospiraceae belonging to Firmicutes, were more abundant in both the DON and ZEN dietary treatments than in the control group. Based on the present data, there may be potential opportunities to isolate and characterize useful probiotics that decrease the level of mycotoxins and help restore intestinal microbiota that have been disturbed by mycotoxins.

## Figures and Tables

**Table 1:** Ingredients and chemical composition of pig standard diet (as-fed basis)

Item	Control diet
Ingredients (%)	
Ground corn	58.56
Soybean meal (46% crude protein)	14.00
Extruded soybean meal	12.00
Whey powder (12% crude protein)	7.00
Fish meal	3.45
Soybean oil	1.60
L-lysine·HCl (78%)	0.43
DL-methionine (99%)	0.14
L-threonine (99%)	0.12
Calcium hydrophosphate	1.08
Limestone	0.60
Choline chloride (50%)	0.20
Sodium chloride	0.32
Vitamin-trace mineral premix1)	0.50
Calculated nutrients (%)	
Metabolizable energy (kcal/kg)	3,444
Crude fiber	2.29
Crude protein	20.78
Crude fat	3.44
Ash	4.35
Lysine	1.47
Methionine	0.49
Calcium	0.75
Phosphorus	0.45

1) Provided the following quantities per kg of complete diet: vitamin, A 11,000 IU; vitamin D_3_, 1,500 IU; vitamin E, 44.1 IU; vitamin K_3_, 4.0 mg; vitamin B_1_, 1.4 mg; vitamin B_2_, 5.22 mg; vitamin B_5_, 20.0 mg; vitamin B_12_, 0.01 mg; niacin, 26.0 mg; pantothenic acid, 14 mg; folic acid, 0.8 mg; biotin, 44 mg; Fe, 100.0 mg as iron sulfate; Cu, 16.50 mg as copper sulfate; Zn, 90.0 mg as zinc sulfate; Mn, 35.0 mg as manganese sulfate; I, 0.30 mg as calcium iodate.

**Table 2:** Relative microbial abundance of cecum major family taxa among control, deoxynivalenol and zearalenone dietary treatment groups

Family name	Percentage of total sequences1)				SEM	p-value

	Collective data2)	Control	DON	ZEN		
Prevotellaceae	21.4	25.4	22.2	17.5	0.088	0.395
Ruminococcaceae	14.7	15.9	13.4	14.9	0.030	0.276
Lachnospiraceae	13.6	14.4	14.1	12.3	0.034	0.309
Veillonellaceae	11.1	9.6	10.6	12.8	0.068	0.623
Bacteroidaceae	4.1	2.0^b^	4.6^a^	5.3^a^	0.064	0.001
Lactobacillaceae	4.0	1.1^b^	6.5^a^	3.8^a^	0.138	0.005
Erysipelotrichaceae	3.8	4.9	2.0	4.7	0.105	0.098
Clostridiaceae	2.0	3.0^a^	0.4^b^	2.7^a^	0.109	0.001
S24–7	1.9	1.6	2.5	1.5	0.120	0.538
Campylobacteraceae	1.4	1.5^a^	0.0^b^	2.8^a^	0.422	0.019
Spirochaetaceae	0.9	0.7	0.8	1.3	0.362	0.796
Coriobacteriaceae	0.8	0.8	0.4	1.2	0.135	0.186
Porphyromonadaceae	0.8	0.5	1.0	0.8	0.109	0.115
Succinivibrionaceae	0.7	0.7	0.6	0.7	0.211	0.887
Aeromonadaceae	0.5	0.2^b^	0.6^a^	0.6^a^	0.062	0.001
Chlamydomonadaceae	0.4	0.2^b^	0.5^a^	0.5^a^	0.069	0.001
F16	0.4	0.4	0.4	0.3	0.295	0.542
Desulfovibrionaceae	0.3	0.3	0.3	0.3	0.130	0.681
Enterobacteriaceae	0.3	0.5	0.2	0.2	0.141	0.996
Pasteurellaceae	0.3	0.4^a^	0.0^b^	0.4^a^	0.394	0.010
Streptococcaceae	0.2	0.3	0.0	0.2	0.260	0.448
Turicibacteraceae	0.2	0.3^a^	0.0^b^	0.2^a^	0.291	0.001
Rhodocyclaceae	0.2	0.1^b^	0.2^a^	0.2^a^	0.173	0.001

DON, deoxynivalenol; ZEN, zearalenone; SEM, standard error of the mean.

1) A total of 192,724 *de novo* OTUs were numbered in serial order.

2) Values represent means.

ab Within a row, different superscript letters indicate a significant difference (p<0.05).

**Table 3:** Diversity statistics of standard diet (control), deoxynivalenol, and zearalenone dietary treatment groups

Sample group	No. of observed OTUs1)	Singles	Doubles	Chao1	Phylogenetic diversity whole tree	Shannon	Simpson group
Control (n = 4)	12,618.50^a^	8,970.00^a^	1,458.25^b^	40,210.65^a^	565.86^a^	8.97^a^	0.98^a^
DON (n = 5)	14,162.60^a^	10,125.80^a^	1,662.40^a^	45,046.49^a^	626.61^a^	9.26^a^	0.98^a^
ZEN (n = 5)	13,519.40^a^	9,582.80^a^	1,599.80^ab^	42,238.81^a^	600.85^a^	9.11^a^	0.98^a^
Pr>F	0.15	0.20	0.05	0.35	0.17	0.35	0.96

OTUs, operational taxonomic units; DON, deoxynivalenol; ZEN, zearalenone.

1) Means among the three dietary groups were compared by analysis of variance, followed by Duncan’s test.

ab Values with different superscript letters in the same column are significantly different (p<0.05).

**Table 4:** Relative microbial abundances of significantly different operational taxonomic units, calculated for the control, deoxynivalenol, and zearalenone dietary treatment groups

OTU ID1)	Classification	Percentage of total sequences2)				SEM	p-value

		Collective data	Control	DON	ZEN		
denovo163744	*Bacteroides*	2.84	1.37^b^	3.12^a^	3.74^a^	0.069	0.002
denovo41137	*Lactobacillus*	1.53	0.25^b^	3.29^a^	0.81^ab^	0.319	0.024
denovo8890	*Campylobacter*	1.04	1.24^a^	0.01^b^	1.92^a^	0.466	0.013
denovo164640	*Prevotella*	0.91	1.79^a^	0.00^b^	1.12^a^	0.593	0.008
denovo3971	Unclassified Clostridiaceae	0.46	1.00^a^	0.06^b^	0.43^a^	0.222	0.002
denovo47258	Unclassified Clostridiaceae	0.46	0.76^a^	0.04^b^	0.63^a^	0.208	0.001
denovo14126	Unclassified Clostridiales	0.45	0.79^a^	0.39^ab^	0.25^b^	0.149	0.048
denovo147371	Unclassified Erysipelotrichacea	0.39	0.67^a^	0.16^ab^	0.41^b^	0.119	0.023
denovo129399	Unclassified Lachnospiraceae	0.33	0.16^b^	0.43^a^	0.37^a^	0.062	0.002
denovo153523	Unclassified Ruminococcaceae	0.29	0.53^a^	0.31^a^	0.10^b^	0.146	0.015
denovo6037	Unclassified Chlamydomonadaceae	0.29	0.13^b^	0.36^a^	0.35^a^	0.070	0.001
denovo24662	Unclassified Lachnospiraceae	0.23	0.25^ab^	0.34^a^	0.10^b^	0.144	0.041
denovo116445	*Bulleidia*	0.23	0.33^a^	0.03^b^	0.35^a^	0.310	0.025
denovo92328	Unclassified Lachnospiraceae	0.22	0.11^b^	0.45^a^	0.09^b^	0.153	0.024
denovo48653	Unclassified Aeromonadaceae	0.22	0.10^b^	0.28^a^	0.25^a^	0.060	0.001
denovo64865	*Bacteroides*	0.22	0.11^b^	0.24^a^	0.28^a^	0.075	0.003
denovo77418	*Paludibacter*	0.18	0.09^b^	0.22^a^	0.21^a^	0.071	0.003

OTUs, operational taxonomic units; DON, deoxynivalenol; ZEN, zearalenone; SEM, standard error of the mean.

1) A total of 52,414 *de novo* OTUs were identified in the three dietary groups in consecutive order.

2) Values indicates the means.

ab Values with different superscript letters in the same row are significantly different (p<0.05).
