# Supplementary material for: Effect of commercially purified deoxynivalenol and zearalenone mycotoxins on microbial diversity of pig cecum contents
Source: Anim Biosci. 2020 May 12;34(2):243–55. doi: 10.5713/ajas.20.0137 (PMC7876725; doi:10.5713/ajas.20.0137)
Supplement: Supplementary file 1 [file ajas-20-0137-suppl.pdf]

Supplementary file 1: The relative abundance of major taxa in three canine breeds

Classification	Percentage of Total Sequences[2]				SEM	*p*-Value	No. of pigs With detect able taxon
	Collective data[1]	Control	DON	ZEN			
**zFirmicutes**	56.8	56.7	54.7	57.8	0.31	0.72	14
Lactobacillus	4.0	1.1[b]	6.5[a]	3.8[a]	0.13	**0.005**	14
Faecalibacterium	4.4	5.5	3.6	4.2	0.08	0.25	14
Dialister	2.3	2.3	1.2	3.4	0.57	0.67	14
Megasphaera	2.0	0.9[b]	3.0[a]	1.8[a]	0.18	**0.05**	14
Lachnospira	1.9	2.5	2.3	1.0	0.17	0.11	14
Phascolarctobacterium	1.7	1.8	1.6	1.7	0.09	0.91	14
Ruminococcus	1.3	1.7	1.1	1.3	0.09	0.14	14
Bulleidia	1.2	1.4	0.8	1.3	0.09	0.38	14
Blautia	1.1	1.2	0.7	1.3	2.2	0.34	14
Anaerovibrio	0.9	0.9	1.2	0.7	0.12	0.15	14
Coprococcus	0.9	1.1	0.8	0.8	0.04	0.15	14
Oscillospira	0.9	0.8	1.1	0.8	0.09	0.44	14
Catenibacterium	0.7	1.0	0.3	0.9	0.38	0.60	13
p-75-a5	0.6	0.7	0.4	0.8	0.28	0.85	12
Clostridium	0.5	0.5	0.2	0.9	0.14	0.11	14
Dorea	0.5	0.4	0.5	0.6	0.08	0.46	14
Megamonas	0.4	0.0	0.0	1.1	0.61	0.36	6
Shuttleworthia	0.3	0.8	0.2	0.1	0.40	0.44	14
Acidaminococcus	0.3	0.3	0.3	0.3	0.43	0.84	14
Butyrivibrio	0.3	0.1	0.1	0.6	0.19	0.18	14
Selenomonas	0.2	0.0	0.5	0.1	0.39	0.38	14
Roseburia	0.2	0.2	0.3	0.1	0.17	0.21	14
Mitsuokella	0.2	0.2	0.2	0.2	0.19	0.89	14
Streptococcus	0.2	0.3	0.0	0.2	0.26	0.40	14
Peptococcus	0.2	0.2[a]	0.0[b]	0.1[b]	0.11	**0.02**	14
Pelosinus	0.2	0.0[b]	0.2[a]	0.1[a]	0.06	**0.005**	14
**Bacteroidetes**	35.6	35.9	38.8	32.0	0.04	0.34	14
Prevotella	21.4	25.4	22.2	17.5	0.09	0.39	14
Bacteroides	4.1	2.0[b]	4.6[a]	5.3[a]	0.06	**0.001**	14
CF231	0.6	0.6	0.7	0.5	0.20	0.67	14
Parabacteroides	0.4	0.3	0.6	0.3	0.22	0.44	14
Paludibacter	0.3	0.2[b]	0.4[a]	0.4[a]	0.07	**0.001**	14
**Proteobacteria**	4.3	4.1	3.1	5.7	0.09	0.23	14
Campylobacter	1.4	1.5[a]	0.0[b]	2.8[a]	0.44	**0.01**	14
Succinivibrio	0.6	0.6	0.6	0.6	0.20	0.81	14

Actinobacillus	0.2	0.1[a]	0.0[b]	0.4[a]	0.38	**0.006**	12
Desulfovibrio	0.2	0.3	0.2	0.2	0.19	0.95	14
Dechloromonas	0.1	0.0[b]	0.2[a]	0.1[a]	0.06	**0.001**	14
**Spirochaetes**	0.9	0.7	0.8	1.3	0.35	0.74	14
Treponema	0.9	0.7	0.8	1.3	0.36	0.79	14
**Cyanobacteria**	0.9	0.7	1.0	0.9	0.08	0.39	14
**Actinobacteria**	0.8	0.8	0.4	1.3	0.29	0.19	14
Collinsella	0.2	0.2	0.1	0.3	0.18	0.24	14
Turicibacter	0.2	0.3[a]	0.0[b]	0.2[a]	0.29	**0.001**	13
**TM7**	0.4	0.4	0.4	0.3	0.26	0.58	14
**Tenericutes**	0.3	0.3	0.4	0.3	0.18	0.71	14
**Verrucomicrobia**	0.2	0.1[b]	0.2[a]	0.2[a]	0.05	**0.001**	14
Akkermansia	0.1	0.0[b]	0.1[a]	0.1[a]	0.04	**0.001**	14
**Fusobacteria**	0.1	0.1	0.1	0.1	0.07	0.41	14

[1] Sequences obtained from all cecum samples

[2] Values represent means.

[a,b] Within a row, means with a different subscript were different ($p < 0.05$).
